# An Audit on the Monitoring and Management of Hyperprolactinemia in Inpatient Adults on Regular Antipsychotics in 8 Acute Wards in an NHS Mental Health Trust

**DOI:** 10.1192/bjo.2022.102

**Published:** 2022-06-20

**Authors:** Peter Zhang, Zhen Dong Li

**Affiliations:** Nottingham Healthcare NHS Foundation Trust, Nottingham, United Kingdom

## Abstract

**Aims:**

The aim of the audit is to ascertain how well hyperprolactinemia is being monitored and managed across all acute adult inpatient wards in a mental health NHS Trust, for patients on regular antipsychotic mediation. The objectives of the audit are to: 1) Assess whether prolactin is being monitored according to local guidelines for patients on regular antipsychotic medication, 2) Determine whether hyperprolactinemia is being identified and managed according to appropriate guidelines, 3) Assess the standard of documentation around the decisions made.

**Methods:**

Data were collected retrospectively from the electronic notes and records for 78 patients, who were discharged from 8 acute wards in February 2021. For checking prolactin test results, the relevant reporting systems were accessed. Two data collection forms were used, which separated patients between those already taking antipsychotics prior to their admission and those who were newly initiated on an antipsychotic.

Patients who were prescribed at least one regular antipsychotic were included. Patients with pre-existing medical conditions that cause hyperprolactinaemia, ongoing pregnancy or breastfeeding were excluded. The monitoring for and management of hyperprolactinaemia was assessed against NICE and local guidance.

**Results:**

From the reviewed data, 41 patients were prescribed at least one regular antipsychotic drug during their admission. 32 patients were already established on an antipsychotic prior to their admission and 9 individuals were started on their first antipsychotic. Hyperprolactinaemia was identified in 9 patients. 19 patients had no prolactin assay performed during their whole admission.

44.4% of antipsychotic naïve patients had a baseline prolactin level taken prior to starting an antipsychotic. 9.1% of patients with hyperprolactinaemia had their symptoms assessed by a clinician. 27.3% of patients with hyperprolactinaemia had actions discussed and undertaken to address this.

**Conclusion:**

This audit identified that patients are at risk of suffering from hyperprolactinaemia and are being insufficiently monitored. The symptoms of hyperprolactinaemia are not adequately screened or assessed for. This may increase the side effect burden and decrease medication adherence among patients. There is a need to increase the awareness among clinicians about the importance of regular prolactin monitoring to improve patient outcomes.

